# Exploring finger millet as an underutilised African indigenous grain crop for functional food development, sustainable nutrition, and health

**DOI:** 10.3389/fnut.2026.1893035

**Published:** 2026-08-19

**Authors:** Mihle Bham, Siphamandla Qhubekani Njabuliso Lamula, Lisa Valencia Buwa, Learnmore Kambizi, Callistus Bvenura

**Affiliations:** 1 Infectious Diseases and Medicinal Plants Research Niche Area, Botany Department, Faculty of Science and Agriculture, University of Fort Hare, Alice, South Africa; 2 Department of Horticultural Sciences, Faculty of Applied Sciences, Cape Peninsula University of Technology, Cape Town, South Africa

**Keywords:** African grains, food security, green revolution, indigenous grains, nutrition security, underutilised grains

## Abstract

**Introduction:**

Food insecurity and malnutrition remain major challenges in Sub-Saharan Africa, driven by climate change and agricultural systems that rely heavily on a few staple crops, including maize, rice, and wheat. The prioritisation of these crops during the Green Revolution has contributed to the neglect of nutrient-rich indigenous grains. Finger millet (*Eleusine coracana*), an indigenous African cereal, has attracted increasing attention because of its exceptional nutritional composition, climate resilience, and potential for functional food development. This review evaluated the role of finger millet in promoting sustainable nutrition, health, and food security.

**Methods:**

A semi-systematic narrative review was conducted following Snyder’s review framework and guided by PRISMA principles for literature identification and screening. Literature published between March 2025 and April 2026 was retrieved from Google Scholar, PubMed, ScienceDirect, Scopus, JSTOR, and ResearchGate using predefined search terms related to finger millet, indigenous grains, nutrition, food security, and functional foods amongst others. An initial 245 articles were identified; after removing 86 duplicates and excluding 12 studies during screening, 147 peer-reviewed articles met the inclusion criteria and were qualitatively synthesised.

**Results:**

The reviewed evidence demonstrates that finger millet is rich in calcium, iron, magnesium, zinc, dietary fibre, and bioactive phytochemicals with antioxidant properties. The crop exhibits excellent drought tolerance and adapts well to marginal environments, making it suitable for climate-resilient agriculture. Finger millet is widely utilised in traditional foods, weaning products, fermented beverages, livestock feed, and value-added products including flour, bakery products, and noodles. However, its wider adoption is constrained by labour-intensive production and post-harvest processing, lower yields than major cereals, pest and weed infestations, limited mechanisation, weak market integration, and inadequate research investment.

**Discussion:**

Finger millet represents a promising underutilised indigenous grain for functional food development, sustainable nutrition, and climate-resilient food systems. Addressing production constraints through improved breeding, mechanisation, research investment, and value-chain development could substantially enhance its contribution to dietary diversification, improved public health, and sustainable food and nutrition security.

## 1 Introduction

The definition of food security has evolved over the past five decades into four interrelated pillars: availability, accessibility, utilisation, and stability. Food security is therefore defined as the availability, accessibility, utilisation, and stability of sufficient, safe, and nutritious food at all times (1). This requires food to be available in adequate quantities, physically and economically accessible, effectively utilised and assimilated by the body, and consistently available to support a healthy and active life (2). In contrast, nutrition security refers to the availability, accessibility, and utilisation of foods that provide all essential nutrients in sufficient quantities to meet daily dietary requirements, thereby supporting growth, physiological functions, immunity, and disease prevention (2).

Food and nutrition insecurity remain major global challenges, with the greatest burden occurring in sub-Saharan Africa (3). The number of hungry people in Africa increased from an estimated 17 million in 2021 to more than 226 million (4), with approximately one in four people experiencing chronic hunger (4). Chronic hunger results from prolonged inadequate dietary energy intake, generally below 1800 kcal per day, the minimum required to sustain a healthy and active life (5).

Historically, food security assessments focused primarily on food availability, often overlooking nutritional quality and contributing to hidden hunger (6). Hidden hunger occurs when sufficient food is available but fails to provide adequate micronutrients. From the 1950s to the early 2000s, agricultural efforts largely prioritised increasing food production rather than improving nutritional quality (7). Consequently, ensuring access to adequate and nutritious food remains a fundamental human right and an urgent global priority.

Children under 5 years of age, women of reproductive age, and older adults are disproportionately affected by food and nutrition insecurity (8). These challenges are further exacerbated by climate variability, economic instability, political factors, and pandemics such as COVID-19, all of which threaten progress towards Sustainable Development Goal 2 (SDG 2), which aims to end hunger by 2030 (3, 9). Although sub-Saharan Africa possesses approximately 60% of the world’s arable land, it contributes only about 10% of global food production (10). This disparity has contributed to a steady rise in undernourishment over the past two decades (11), underscoring the need for interventions that improve food availability, accessibility, and nutritional quality.

The underutilisation of Africa’s agricultural potential highlights the need to integrate indigenous knowledge systems (IKS), including underutilised indigenous grains, into food and nutrition security strategies. These crops offer considerable potential to alleviate food shortages, reduce micronutrient deficiencies, and enhance agricultural diversity (12). Despite their abundance across Africa, indigenous grains remain largely neglected. Compared with major cereals such as maize, rice, and wheat, many indigenous grains exhibit greater tolerance to drought, diseases, and poor soils (13). This review therefore examines the potential contribution of African underutilised indigenous grains to food and nutrition security, with particular emphasis on finger millet (*Eleusine coracana*).

Several recent reviews have comprehensively examined the nutritional composition, bioactive compounds, and processing characteristics of finger millet (14–17). However, two important gaps remain. First, most reviews present finger millet’s nutritional and health benefits as broadly applicable without adequately considering the African historical and food systems context. Although finger millet originated in Africa (18), it was marginalised, together with other indigenous grain crops, during the Green Revolution. This review places finger millet within that historical context, linking its continued underutilisation to agricultural research priorities and crop investment patterns rather than treating its neglect as incidental.

Second, previous reviews often synthesise evidence from *in vitro*, animal, and human studies without distinguishing the relative strength of evidence. This review explicitly differentiates mechanistic findings from clinical evidence, highlighting where promising laboratory results, such as enzyme inhibition assays, have yet to be confirmed by adequately powered human studies. It also critically evaluates the quality of available production statistics, demonstrating that FAO data aggregates all millet species, thereby obscuring finger millet-specific production trends and the identity of the leading producer. Finally, this review presents an integrated conceptual framework linking climate change, sustainable agriculture, finger millet production, nutritional benefits, functional food development, and health outcomes as interconnected components of a single system. Collectively, these perspectives address not only current knowledge on finger millet but also the evidentiary, statistical, and contextual gaps that remain insufficiently addressed in the existing literature.

## 2 Literature search methodology

### 2.1 Review design

This review follows a semi-systematic (narrative) methodology using the approach outlined by Snyder (19), rather than a fully systematic or meta-analysis review. This method was selected because the literature on finger millet as a functional food and nutritional source is methodologically heterogenous, spanning agronomy, food science, nutrition, and human health and therefore it is not well suited for the narrow, homogeneous outcome pooling required by the PRISMA style systematic or meta-analysis review. A semi systematic or narrative review allows for a transparent, reproducible search and selection process whilst maintaining the flexibility to synthesise qualitative and quantitative evidence across disciplinary boundaries.

### 2.2 Search strategy

A thorough literature search was conducted between March 2025 and April 2026 using search engines such as Google scholar, PubMed, Science Direct, Scopus, JSTOR, and Research following PRISMA (Preferred Reporting Items for Systematic Reviews and Meta-Analyses) guidelines gate to gather documented information on finger millet. The PRISMA guidelines were adopted to ensure rigorous and transparent information gathering. The focus was on finger millet phytochemistry, biological activities, as well as cultivation practises and its potential contribution to mitigating food insecurity and malnutrition, as well as its health benefits. The search terms included “food insecurity,” “Green Revolution,” “malnutrition,” “undernourishment,” “indigenous grains,” “underutilised grains,” “millets,” “finger millet,” and “*Eleusine coracana*.”

### 2.3 Eligibility criteria: inclusion and exclusion criteria

The screening process was conducted in a structured and transparent manner to ensure that only relevant and methodological studies were included in the review (19). Initially, all retrieved articles were screened by reading their titles and abstracts. Studies that were clearly unrelated to indigenous African grains, millets, finger millet, *Eleusine coracana,* food security, food insecurity, nutrition, malnutrition, or indigenous knowledge systems were excluded at this stage. Duplicate records identified across databases were removed prior to further screening.

**Figure 1:**
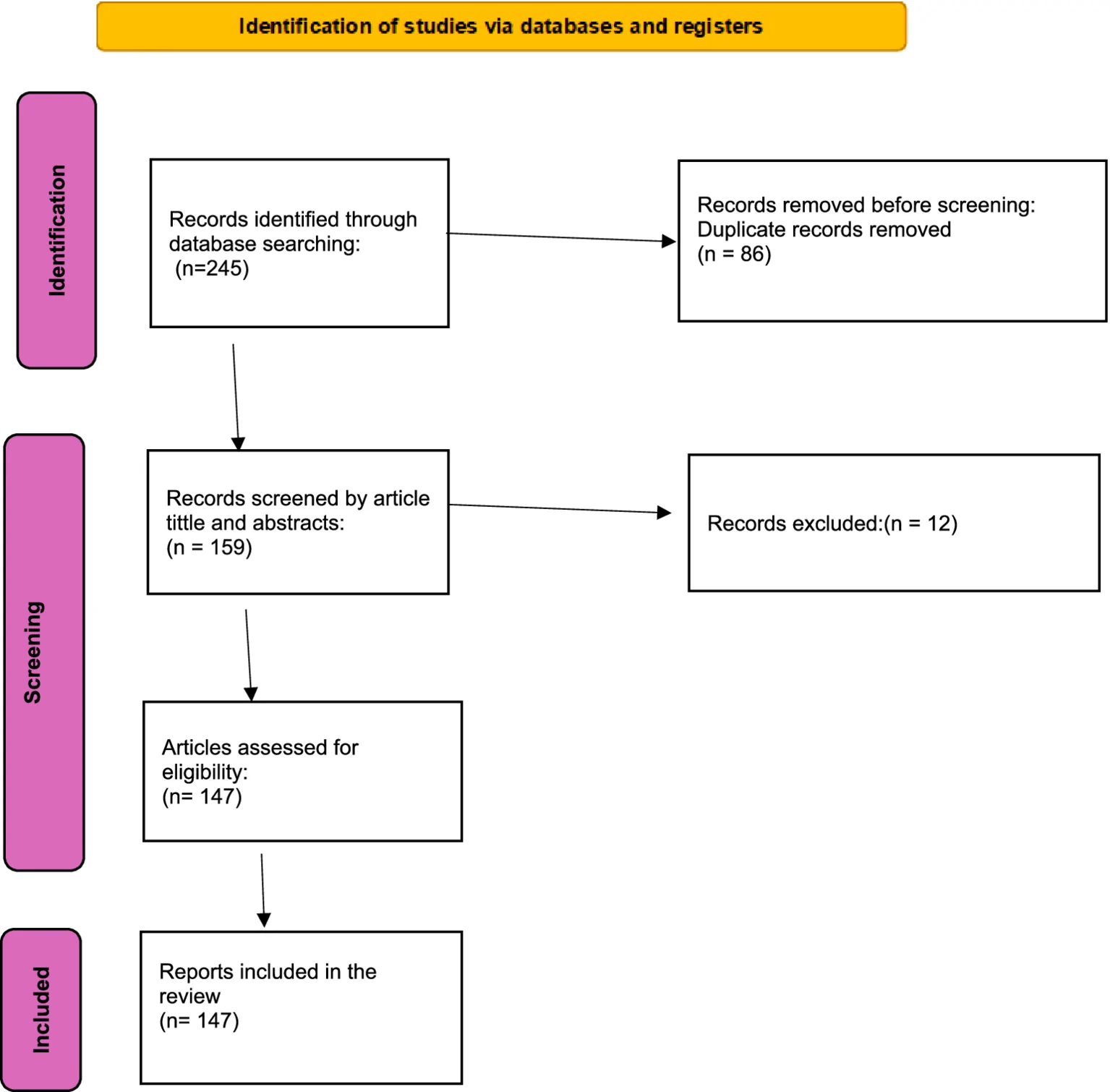
Illustrates the literature search and data search compilation detailing the number of screened, excluded and included reports for the review.

Following the initial screening, full-text articles were assessed for eligibility. Studies were excluded if they did not directly address the research objectives, lacked sufficient methodological detail, or presented inadequate sample sizes. Non-peer-reviewed publications, opinion pieces, thesis, and editorial articles were also excluded to maintain scientific rigour. The reviewer workflow involved a systematic and consistent evaluation of each study against predefined inclusion and exclusion criteria. Only studies focusing on African indigenous and underutilised grain species were considered, with particular emphasis on nutritional compositions, bioactive compounds, and their role in addressing food insecurity and malnutrition. Studies investigating cultivation practises, processing methods, and utilisation of finger millet and related grains were also included. The selection process was conducted as follows: the initial search resulted in 245 articles. After deleting 86 duplicates, 159 articles were screened using the title and abstract and 12 articles were removed because they did not meet fit the inclusion requirements. A total of 147 articles were evaluated and included in the review. Figure 1 simplifies this data by presenting a complete diagram of the entire selection process.

### 2.4 Review framework and scope

To ensure conceptual clarity and alignment of the included evidence, this review was driven by a modified Population Intervention Comparator Outcomes Study design (PICOS) framework. Since the review synthesised several study designs, no explicit comparison was needed. Adoption of this paradigm gave topic synthesis and literature selection an organised foundation. Conclusions about nutritional and functional benefits are solely based on peer-reviewed experimental results and traditional practises are presented to provide cultural and historical context.

## 3 Climate change, food insecurity, and the need for resilient indigenous crops

Climate change is one of the leading drivers of food and nutrition insecurity in Africa. Its impacts, including prolonged droughts, rising temperatures, and increasingly variable rainfall patterns, have significantly reduced agricultural productivity (20). This is particularly evident in southern Africa, where agriculture is predominantly rainfed (21). Global warming is projected to increase undernourishment in sub-Saharan Africa by 25–90% by 2050 (22, 23). For example, a study conducted in Niger reported an association between maternal and neonatal mortality, with poor nutrition identified as a contributing factor (24). These findings highlight the need for policies that strengthen nutrition under changing climatic conditions. At the same time, continued population growth will increase the demand for food (25). Together, climate change, population growth, and economic instability underscore the urgent need to develop sustainable food systems that harness local resources and indigenous knowledge to increase the production of nutritious foods.

In southern Africa, drought and rising temperatures have substantially reduced agricultural production. Approximately 95% of farming in the region is rainfed, making it highly vulnerable to climate variability and exacerbating food insecurity (21). Declining agricultural productivity, coupled with socio-economic challenges such as unemployment, perpetuates poverty and limits the capacity of smallholder farmers to adapt to changing climatic conditions (26, 27). Women and children are particularly vulnerable because they already experience disproportionately high rates of undernutrition and micronutrient deficiencies (28). Consequently, climate change reduces food availability, increases malnutrition, worsens health outcomes, lowers labour productivity, and weakens household and national economies (29). Although climate-smart agriculture, including practises such as intercropping, offers promising adaptation strategies, implementation remains limited. These challenges highlight the need to complement existing interventions by promoting African underutilised indigenous grains as climate-resilient crops capable of strengthening food and nutrition security.

Understanding this vulnerability requires examining the agricultural policies that shaped crop production in Africa. Agriculture aims to provide sufficient, safe, and nutritious food that meets dietary needs and preferences for a healthy and active life, yet this objective remains elusive across much of the continent (30). Since the Green Revolution began in the 1940s, global food production has consistently outpaced population growth (31). The Green Revolution was characterised by the widespread adoption of high-yielding crop varieties, mechanisation, and chemical fertilisers, resulting in increased crop yields and shorter production cycles (31). However, these gains were driven primarily by investments in rice, wheat, and maize and were far less evident in Africa.

Although approximately 30,000 edible crop species exist, only about five currently provide most of the world’s food supply, including the major cereals prioritised during the Green Revolution (18, 147). These staple crops contribute approximately 40–60% of global dietary energy intake (156). Rice (*Oryza sativa* L.) was domesticated in China about 9,000 years ago (32) and, although its introduction into Africa remains debated, Kehinde (33) suggests it was brought to West Africa by the Portuguese around 1,500 AD. Wheat (*Triticum aestivum* L.) originated in southwestern Asia around 10,000 BC (34) and was introduced into Africa approximately 90 years ago (18). Maize (*Zea mays* L.), domesticated about 9,000 years ago in southwestern Mexico (35), reached Africa approximately 500 years ago during the Columbian Exchange (18).

According to Pingali (36), these crops received substantial research investment and institutional support in Japan, the United States, and Europe, driving remarkable production increases of 108, 208, and 157% for rice, wheat, and maize, respectively. However, these gains largely excluded Africa. This disparity may partly reflect limited investment in indigenous African crops, whilst research and development focused predominantly on species originating outside the continent. Consequently, indigenous grains were progressively neglected, and their cultivation declined (18), despite having been domesticated in Africa long before the Green Revolution (Table 1).

**Table 1:** An overview of millets.

Type of millet	Botanical name	Origin	Domestication period	Major producers	Nutritional constituents	Other uses	References
Finger millet	*Eleusine coracana* (L.) Gaertn.	Western Uganda and Ethiopia	~3,000 BC	Uganda, Ethiopia, Tanzania, India, Sri Lanka, China, Taiwan, Indonesia, and Guam	Iron, protein, calcium, fibre, vitamins, and low glycaemic index	Alcohol production, livestock feed, and construction	(54, 62, 122, 129)
Proso millet	*Panicum miliaceum* L.	Northern China	8,350–6,750 BC	China, India, Nepal, Kenya, Ethiopia, Russia, Ukraine, and Belarus	Carbohydrates and protein	Animal feed	(49)
Foxtail millet	*Setaria italica* (L.) P. Beauv.	Northern China	8,050–7,050 BC	China, Ukraine, United States, Australia, and Egypt	Essential amino acids	Bird feed and fodder	(131)
Kodo millet	*Paspalum scrobiculatum* L.	India	~977 BC	China, India, Russia, and Japan	Fibre and vitamins	Fodder and Cover crop	(132)
Pearl millet	*Pennisetum glaucum* (L.) R. Br.	Northern Mali	~5,000 BC	Nigeria, Mali, India (largest producer), Niger (staple)	Rich in minerals, B vitamins, and fatty acids	Poultry feed and alcohol production	(129)

Because human diets depend largely on available crops, the emphasis on a few staple cereals reduced agricultural diversity and dietary diversity (37). Although the Green Revolution substantially improved food availability, reliance on a limited number of staple crops has been associated with micronutrient deficiencies, malnutrition, and an increasing prevalence of non-communicable diseases (38). Diversifying crop production has been shown to improve dietary diversity and micronutrient intake, thereby enhancing nutritional adequacy (39, 40).

The intensive use of chemical fertilisers to sustain high crop yields has also negatively affected soil health by reducing microbial diversity, altering soil pH, decreasing soil porosity, and contributing to water contamination (41). Likewise, monoculture-based farming systems, another defining feature of the Green Revolution, lack the genetic diversity needed to adapt to changing environmental conditions, making them increasingly vulnerable to climate change (42). These limitations emphasise the need to integrate locally available, nutrient-rich crops into mainstream agriculture. Underutilised indigenous grains offer considerable potential to complement conventional cereals whilst enhancing dietary quality, increasing agricultural biodiversity, improving soil health, strengthening ecosystem resilience, and promoting more climate-resilient farming systems.

Africa possesses a rich diversity of indigenous grain crops with considerable potential to strengthen food and nutrition security. Underutilised crops are species that were once widely cultivated within their native communities but have gradually declined in use despite their nutritional value, adaptability, and health benefits (43, 44). Often referred to as orphan, neglected, or lost crops, many have experienced a steady decline in cultivation since the Green Revolution (45). Their prolonged neglect has resulted in many being regarded as wild species, weeds, or “poor man’s foods” (46). However, growing concerns over food and nutrition insecurity have renewed interest in these crops because of their potential to alleviate hunger, improve dietary quality, and enhance agricultural resilience (47). Today, they continue to be cultivated primarily by smallholder farmers in resource-poor communities (48).

## 4 Millets: diversity, domestication, and nutritional composition

Millet species, including finger millet (*Eleusine coracana* (L.) Gaertn.), proso millet (*Panicum miliaceum* L.), foxtail millet (*Setaria italica* (L.) P. Beauv.), kodo millet (*Paspalum scrobiculatum* L.), and pearl millet (*Pennisetum glaucum* (L.) R. Br.), represent a diverse group of ancient crops with origins spanning across Africa and Asia (49). As shown in Table 1, the domestication of these grains started millennia ago in various parts of the world and are currently cultivated across various parts of the world. Their cultivation is more prevalent in semi-arid and marginal environments (49). However, despite their wide global distribution across different regions, their global production remains extremely low when compared with conventional crops such as rice and maize (18).

As shown in Table 1, millets are multifunctional crops that, in addition to supporting human nutrition, are used in the production of traditional fermented alcoholic beverages, the production of animal feed, and can be used as cover crops to improve soil health and promote environmental sustainability (50). All these applications further emphasise the significance of millets in human livelihoods. Due to their adaptability to harsh climatic conditions, tolerance of poor soils, and high nutritional value, these grains have considerable potential to improve food and nutrition security. However, comprehensive research on individual millet species is needed to better understand their roles in sustainable agricultural systems, their contributions to future food and nutrition security strategies, and their associated broader health benefits. According to Singh et al. (49), millets are a group of small-seeded cereal crops belonging to the Poaceae family, characterised by their distinctive inflorescences and unique morphological, physiological, and nutritional attributes. They were domesticated independently in several regions of the world, including East Asia, South Asia, West Africa, and East Africa, with proso millet and foxtail millet recognised as the earliest domesticated millets in China approximately 10,000 years ago (18). Archaeological evidence also indicates that several millet species were domesticated in Africa, where they have been cultivated since approximately 4,500 to 5,000 and have long served as important food crops (51, 52) (151).

Today, millets are widely cultivated in countries such as India, Niger, Sudan, Nigeria, and Chad, occupying approximately 32.2 million hectares globally (53). In southern Africa, they are predominantly produced by smallholder farmers in countries including South Africa, Zambia, and Zimbabwe, where they remain an important component of traditional farming systems (18). These indigenous and underutilised cereals play a significant role in supporting the livelihoods and food security of rural and marginalised communities, many of whom depend on them as a major source of dietary energy and essential nutrients (48). In regions where millets are staple foods, they constitute a substantial proportion of daily dietary intake. Amongst the various millet species, finger millet is particularly noteworthy because of its exceptional tolerance to drought, poor soils, and other environmental stresses, together with its distinctive nutritional constituents and functional properties (18). These attributes make it one of the most promising climate-resilient crops for enhancing sustainable agriculture. In addition, millets are excellent sources of minerals and vitamins (Table 2) as well as proximate constituents (Table 3), making them valuable for addressing malnutrition and micronutrient deficiencies (43).

**Table 2:** Comparative mineral and vitamin content of different types of millets and maize.

Minerals (mg/100 g)	Finger millet	Pearl millet	Foxtail millet	Kodo millet	Proso millet	Little millet	Maize
Ca	240–344	16	–	15.27	8–20.0	16.06	7
Fe	1.90–6.30	6	2.90	0.90	2.34	1.26	2.71
Zn	1.95–4.27	3.40	2.50	–	1.50	1.82	2.21
Mg	130–188.83	228	82	122	154	91.41	127
K	350–500	390	–	144	–	–	286
Riboflavin	19–0.33	5.80	0.20	0.20	0.40	1.29	0.08
Thiamine	0.48	8.42	0.70	0.29	0.50	0.26	0.385
Niacin	–	9.40	–	1.49	1.55–3.70	0.05	1.90
References	(130)	(133)	(45, 134)	(135)	(45)	(136)	(130)

**Table 3:** Proximate compositions of different millets and maize.

Grain type	Carbohydrates (%)	Proteins (%)	Dietary fibre (%)	Fat (%)	References
Finger millet	72.6	3.49–13	3.6–11.5	1–2	(130)
Pearl millet	67.5	11.6	1.2	3–5	(133)
Foxtail millet	60.9	12.3	19.1	4.3	(134, 152)
Kodo millet	66.19	8.92	37.8	1.4	(135)
Proso millet	70.4	11	2–7	3.5	(152)
Little millet	73.40	10.13	7.72	3.89	(136)
Maize	74.3	9.42	7.3	3.29–4.25	(130)

## 5 Finger millet: origin, taxonomy, morphology, ecology, and agronomy

Despite its long history of cultivation, the importance of finger millet has declined over time because of the global dominance of the major staple cereals (18). Nevertheless, its exceptional adaptability to marginal environments and high nutritional value have renewed interest in the crop as a sustainable solution to food and nutrition insecurity.

Non-the-less, this crop belongs to the family Poaceae and is taxonomically distinct from most cultivated millets, as it is classified within the subfamily *Chloridoideae* and tribe *Chlorideae*, whereas most other millets belong to the subfamily *Panicoideae* (54). It is the fourth most important millet globally after sorghum, pearl millet, and foxtail millet (55). The crop is predominantly self-pollinating, with only about 1% natural cross-pollination occurring through wind. It is a tetraploid species (2n = 4x = 36) believed to have evolved from its wild progenitor, *Eleusine africana* (54).

Finger millet is a fast-growing C₄ annual grass that matures within 3–6 months and typically reaches 30–150 cm in height, although plants may grow up to 170 cm under favourable conditions (56). It produces long, narrow leaves, and its relatively small leaf area contributes to reduced water loss and improved adaptation to high-temperature environments. The crop performs well at elevations between 1,000 and 2,000 m above sea level in eastern and southern Africa (57). It grows optimally at approximately 23 °C but tolerates temperatures ranging from 11 to 35 °C and performs best under seasonal rainfall of 500–1,000 mm. Although widely recognised for its drought tolerance, prolonged intermittent or terminal drought can still significantly reduce grain yield (58).

**Figure 2:**
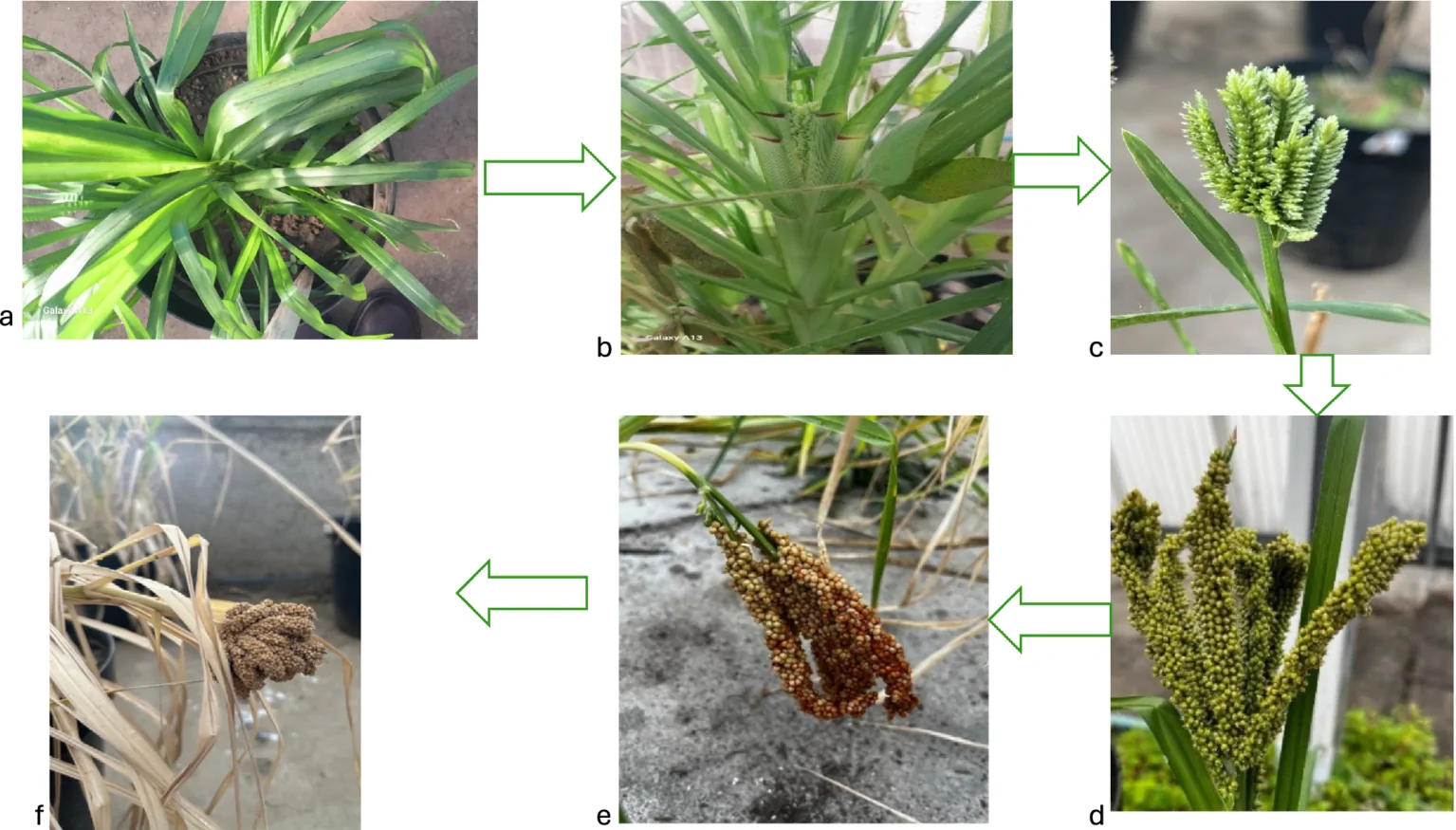
Finger millet plant at different maturity stages from a green healthy growing plant to a mature brown plant. The common name “finger millet” is derived from its distinctive digitate inflorescence, in which the spikes resemble the fingers of a hand (129). As illustrated in Figure 1, exhibits six distinct developmental stages of finger millet: **(a)** the vegetative stage, marked by tillering and leaf development at the base of the plant, progressing through **(b)** stem elongation and booting, during which the panicle begins to develop within the leaf sheaths. **(c)** This is followed by the panicle emergence and inflorescence development where the characteristic finger-like spikes forms and expand whilst still green. **(d)** The grain filling stage is characterised by a fully emerged green panicle head with developed grains. **(e)** The final stages of grain development and ripening as the fingers fill with grain and shift from green to a reddish-brown hue during grain-filling resulting in **(f)** full physiological maturity, when the panicle and surrounding foliage dry out and turn straw-coloured, signalling readiness for harvest. Finger millet is also known by numerous local names, reflecting its wide geographical distribution and cultural importance. These include African millet (England), ragi/mandua (India), korakan (France), bulo (Uganda), dagusa/tokuso (Ethiopia), wimbi (East Africa – Swahili), telebun (Sudan), uphoko/rapoko/zviyo/njera/rukweza (Zimbabwe) or telebun in the Sudan (60, 130).

The ability of finger millet to produce relatively stable yields under marginal environmental conditions, combined with its exceptional nutritional value, makes it a promising crop for climate-resilient and sustainable agriculture. Fully exploiting this potential, however, requires a comprehensive understanding of its cultivation requirements and agronomic management practises that maximise productivity across diverse agroecological conditions (Figure 2).

### 5.1 Cultivation and agronomic practises

The productivity and resilience of finger millet are influenced by several agronomic factors, including site selection, planting time, nutrient management, weed control, and harvesting practises. Table 4 summarises the recommended cultivation and agronomic practises for finger millet, adapted from Praveen et al. (59).

**Table 4:** Finger millet cultivation and agronomic practises.

Cultivation/agronomic practise	Description
Soil	Finger millet can grow under different soil conditions. It can withstand infertile and mild alkaline soils. However, it grows best in alluvial, loamy, and sandy soils with good drainage.
Land preparation	Months prior to cultivation it is most advisable to prepare the land by performing one deep ploughing with a mouldboard. Secondary tillage with a cultivator and multiple tooth hoe is essential for smooth seedbed preparation.
Seed rate, spacing, and sowing time	The recommended seed rate for sowing a one-hectare field is 8–10 kg of seed. Even though random broadcasting is usually used for finger cultivation, to achieve best plant population line sowing at a spacing of 22.5–30 cm between rows and 7.5–10 cm between plants and 2–4 cm of sowing depth is recommended. Additionally for an ideal finger millet population, a 4–5 lakh plants/ha must be maintained. It is usually cultivated during the rainy season of the year.
Nutrient management	An application of 5–10 t/ha farmyard manure a month before sowing is advisable to hold moisture content in the soil. Synthetic fertiliser recommendations are 40 kg nitrogen (N), 20 kg phosphorus (P), and 20 kg potassium(K) per ha for rainfed cultivation and 60 kg N, 30 kg P, and 30 kg K per ha for irrigation.
irrigation management	Finger millet is usually cultivated in rainy season therefore it normally does not require any irrigation process. However, if the rainfall becomes scarce, irrigation may be introduced especially in critical stages of growth of the plant such as tillering and flowering stages. This crop also cannot survive in waterlogged areas, therefore should there be heavy rains and there is excess water in the soil in needs to be removed
Harvesting and post-harvest management	When finger reaches its full physiological maturity, it must be harvested and because of rapid changes in temperatures and humidity content late harvesting may result in yield losses. Harvesting can either be done with hands or using machinery by cutting panicles and threshing them using sticks. When harvesting moisture content should be thoroughly monitored, harvesting when grain content is at 16–20%. Then grain moisture is further dried to about 13% before they are stored. Proper drying, handling, and storage are essential to prevent insect infestation, mould development, and post-harvest losses.

Adapted from Praveen et al. (59).

## 6 Constraints to production, commercialisation, and value chains

The successful cultivation of finger millet is often constrained by a combination of biotic, abiotic, agronomic, and socio-economic factors.

### 6.1 Socio-economic constraints

Finger millet production remains low in Africa despite its value as a staple crop in some regions. For instance, farm yields in Uganda are less than 1,000 kg/ha compared to potential yields of up to 5,000 kg/ha under research conditions (60). This large gap yield is mostly determined or influenced by socio economic constraints such as low yielding unimproved varieties, poor agronomic practises, and inadequate post-harvest handling of the grains (60). Smallholder farmers often exchange seed varieties informally without prior improvement or evaluation, primarily for sharing purposes. This results in continued use of traditional seeds which are often associated with losses (61). Consequently, limited research is conducted on the crops they cultivate, as production practises rely on traditional knowledge passed down across generations, despite changing environmental conditions. Finger millet is a labour-intensive crop, however its production lacks machinery as it is mostly cultivated by small holder farmers (62). This hinders the expansion of small holder farmers who cultivate it. Also, finger millet is categorised as an underutilised crop since it has been receiving little attention and research when compared to other grains such as maize, thereby limiting improvement on its cultivation area. However, finger millet possesses high adaptability characteristics to harsh environmental conditions suggesting that addressing these socio-economic constraints could significantly enhance productivity and contribute to food security (63).

### 6.2 Finger millet value-chain, market, and institutional constraints to African production and commercialisation

The finger millet market is often concentrated to certain regions and population groups such as parts of Kenya’s Eastern and Nyanza regions, Tanzania’s central and northern zones, Uganda’s eastern and northern areas and is not broadly commercialised like rice and maize which can be found almost in all parts of a country. In Zimbabwe, it is sparsely distributed throughout the Matabeleland and Midlands provinces, and parts of Mashonaland provinces. However, in South Africa, production is concentrated in the Venda speaking regions of the Limpopo province and virtually forgotten in the remaining regions. A study focussing on the consumption of finger millet in these countries revealed that the crop is mostly consumed in rural areas when compared to urban areas (18). Additionally, with an increase in income, there is also an increase in finger millet consumption, however per capita consumption of finger millet remains lower than that of wheat and maize (64). As a result, the relatively limited market demand reinforces existing value-chain constraints, reducing incentives for producers, processors and other stakeholders to invest in long-term commercial relationships.

A survey of 53 sorghum and finger millet processing firms in Kenya, Tanzania, and Uganda, three of Africa’s leading producers, found that processing is dominated by small- and medium-scale enterprises with limited capacity to absorb price fluctuations or invest in improved equipment and machinery (65). Most firms employed between eight and ten workers and had an annual grain demand of less than 50 tonnes, with Kenya being the exception, where annual demand exceeded 600 tonnes (65). The study also revealed weak linkages between producers and processors. Whilst approximately two-thirds of processors in Kenya and one-third in Tanzania and Uganda sourced grain directly from farmers, challenges related to contract enforcement and transport often forced them to rely on traders instead (65).

These value-chain challenges are compounded by weaknesses in seed systems, farmer organisation, and access to production resources. Formal finger millet seed systems remain poorly developed compared with those of major staple crops, leaving most farmers dependent on informal seed networks. A study involving 1,001 rural households in Kenya, Tanzania, and Uganda found significant gender disparities in access to extension services, farmer groups, and farmer-to-farmer seed exchange networks amongst producers of beans, finger millet, and sorghum (66). Women had fewer links to formal extension services and input suppliers than men, despite playing a central role in finger millet production and processing. These institutional inequalities limit access to improved seed, technical knowledge, and production inputs, ultimately constraining crop productivity.

Weak farmer-processor linkages, underdeveloped seed systems, and limited collective marketing infrastructure constitute the major constraints to finger millet commercialisation. These challenges are closely interconnected. Poor seed systems and inadequate institutional support reduce productivity and grain quality, resulting in inconsistent supplies that weaken relationships between farmers and processors. Consequently, processors increasingly depend on intermediaries, whilst limited marketing channels contribute to unstable markets and high grain prices. Addressing these constraints therefore requires integrated interventions that simultaneously strengthen seed systems, farmer organisations, market linkages, and value-chain infrastructure. An additional challenge is the geographic concentration of finger millet production within relatively few regions, which has contributed to limited research investment and hindered its integration into mainstream food systems.

### 6.3 Biotic and abiotic constraints

In addition to socioeconomic constraints, production of finger millet is also negatively affected by biotic and abiotic factors. These significantly affect crop production by determining plant health, growth rates, and overall yield (67). Biotic factors include diseases, weeds, and insects whilst abiotic factors include drought.

#### 6.3.1 Biotic stressors

##### 6.3.1.1 Blast disease

Blast disease, caused by the fungus *Pyricularia grisea* (teleomorph: *Magnaporthe grisea*), is the most economically important biotic constraint to finger millet production. The disease causes yield losses of 50–90% in Uganda and approximately 16% in western Ethiopia. It also infects other cereal crops, including wheat, rice, pearl millet, and foxtail millet, resulting in substantial yield losses across Africa and Asia (60). The pathogen attacks all above-ground parts of the plant throughout its growth cycle. Leaf symptoms are characterised by grey, elliptical or diamond-shaped lesions that reduce photosynthetic capacity, impair physiological development, and ultimately decrease grain yield. The fungus damages the parenchymatous, sclerenchymatous, and vascular tissues, restricting the translocation of nutrients to developing grains, thereby reducing grain formation. Seed infection further lowers germination, compromising subsequent crop establishment (68).

Management of blast disease includes cultural practises such as early planting, effective weed control, and crop rotation, although these measures alone rarely provide adequate control. Fungicides, including pyroquilon, tricyclazole, mancozeb, and carbofuran, have proven effective; however, their high-cost limits adoption by resource-poor smallholder farmers, and their widespread use raises environmental concerns. Consequently, the development and cultivation of blast-resistant finger millet varieties, such as NAROMIL 1–5 and SEREMI 2, combined with appropriate agronomic practises, have been proposed as a more sustainable and economically viable management strategy (60).

##### 6.3.1.2 Weeds

Weeds are amongst the major constraints affecting finger millet production, with grass weeds posing the greatest challenge because of their morphological similarity to finger millet. This problem is particularly prevalent in broadcast cultivation systems, where distinguishing between the crop and weeds during early growth stages is difficult (69). In addition, parasitic weeds belonging to the genus *Striga* (family Orobanchaceae) are highly destructive, attaching to finger millet roots and remaining difficult to detect until significant damage has occurred (70). *Striga hermonthica* (Del.) Benth. is one of the most damaging species affecting finger millet, causing substantial yield reductions and increasing labour requirements for control measures (71). Because *Striga species* establish underground on host roots, infestations can remain undetected and spread across multiple farms before effective management is implemented (72). In Ethiopia, *Strig*a accounts for approximately 55% of the production constraints experienced by finger millet farmers (60, 73). Therefore, promoting access to improved resistant varieties, such as the *Striga*-resistant Baruda variety developed in Ethiopia, is essential for reducing losses amongst smallholder farmers (60).

##### 6.3.1.3 Pests and bugs

Several insect pests affect finger millet production, with stem borers and ragi aphids amongst the most destructive. Stem borers, particularly the pink stem borer (Sesamia inferens), cause damage from the seedling stage through to grain maturity (74). The larvae enter the leaf whorl and feed on soft tissues, producing characteristic pinhole damage that becomes visible when affected leaves unfold. As larvae tunnel into the stems, they disrupt vascular tissues, resulting in drying of the central shoot during the vegetative growth stage and ultimately reducing crop productivity (74).

**Figure 3:**
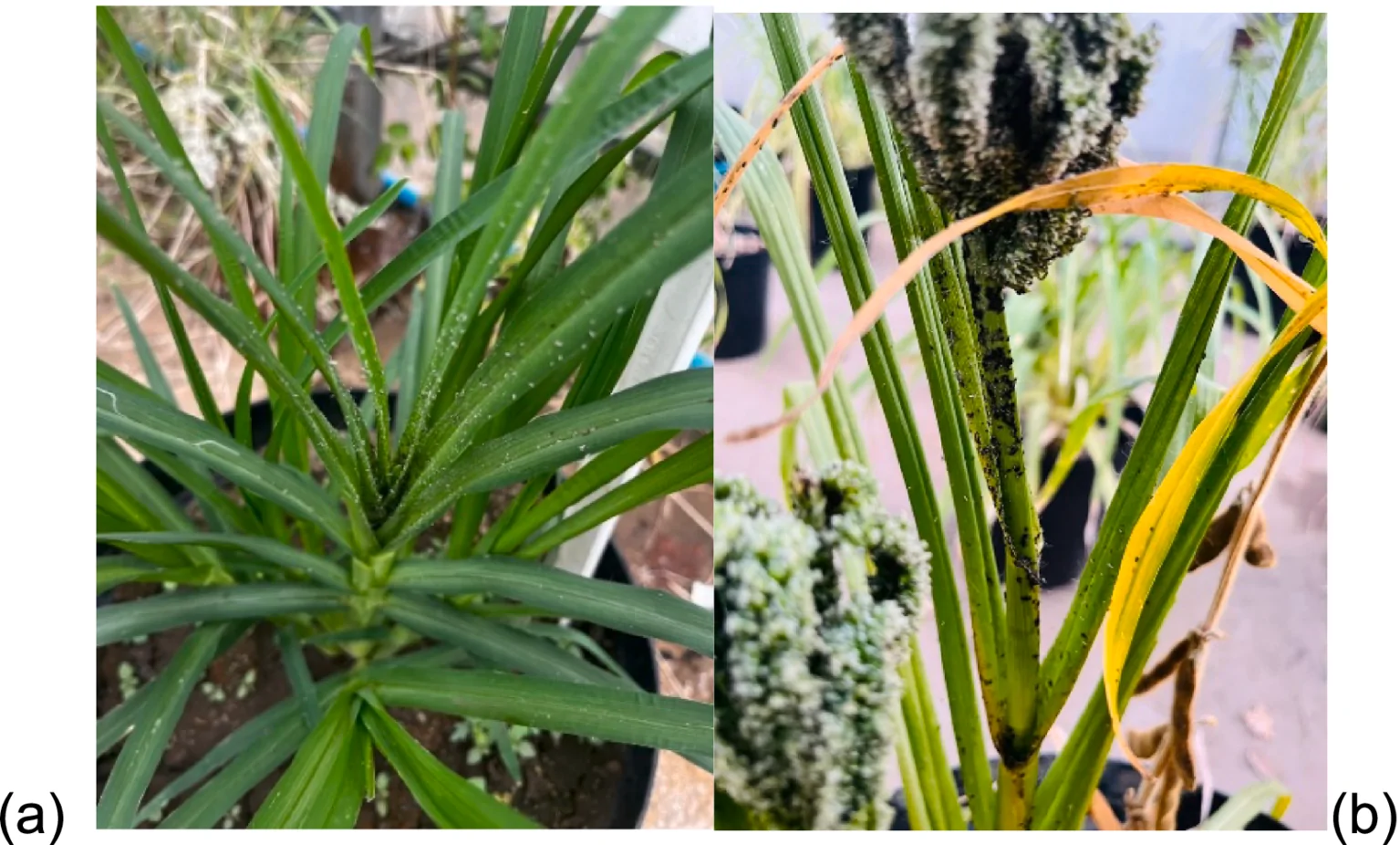
Finger millet plants cultivated under glasshouse conditions infested with aphids **(a)** early infestation during the vegetative growth phase with minimal visible folia damage, and **(b)** advanced sustained infestation at the reproductive stage showing dense aphid colonies around the peduncle and leaf sheath with premature leaf yellowing.

The ragi aphid (Tetraneura nigriabdominalis) is another important pest belonging to the family Aphididae. It colonises finger millet roots near the collar region (Figure 3) and is frequently associated with ants (75). This association results from a mutualistic relationship in which aphids provide ants with honeydew, whilst ants offer protection, feeding assistance, and transportation to aphids (76). Ragi aphids are typically globular or oval-shaped and vary in colour from green to yellow or brown (Figure 2). Following infestation, they reach maturity within 7–9 days, with adults having a lifespan of approximately 5–11 days and exhibiting both sexual and asexual reproductive capacity (76). Their ability to produce 10–35 offspring per reproductive cycle contributes to rapid population increase once established (75). By sucking plant sap, aphids weaken plants, causing stunted growth, wilting, and eventual drying of the crop (77).

#### 6.3.2 Abiotic stressors

##### 6.3.2.1 Drought

Finger millet is predominantly cultivated under semi-arid and arid conditions; however, drought and inadequate soil moisture remain major abiotic constraints limiting its productivity. These stresses negatively affect crop growth, development, and yield potential. Stress tolerance varies amongst finger millet varieties, with brown finger millet demonstrating greater drought tolerance than black finger millet (78). The crop is particularly vulnerable to terminal drought, which occurs when water becomes limited towards the end of the growing season during grain filling and maturity stages (79, 154). This poses a significant challenge for smallholder farmers who largely depend on rainfall for crop production.

Drought stress reduces plant growth by limiting cell expansion and elongation, resulting in reduced biomass accumulation and grain yield. It also decreases seed weight, chlorophyll content, and leaf area, thereby affecting photosynthetic capacity and overall crop performance (60). These effects significantly constrain productivity, particularly amongst subsistence farmers operating under rainfed conditions.

Socio-economic constraints, biotic stresses, and abiotic stresses collectively limit finger millet production and commercialisation despite the crop’s nutritional and agronomic value. Although various management strategies, including traditional practises and chemical interventions, have been developed to address these challenges, traditional approaches are often labour intensive, whilst chemical inputs are costly for resource-limited farmers and may negatively affect soil health when overused. Therefore, the development and promotion of improved finger millet varieties with enhanced resistance to major production constraints are essential for achieving sustainable production, improving farmer livelihoods, and strengthening food and nutrition security.

## 7 Production status and economic importance

The widespread cultivation of finger millet in parts of Africa, particularly East Africa, contributes significantly to household food security and income generation. The crop serves as both a staple food and a cash crop, with surplus grain after household consumption commonly sold through local markets and community networks (80). Finger millet grains also have excellent storage characteristics, remaining viable for 1–2 years, particularly under low-humidity conditions and temperatures below 20 °C. Its natural resistance to pests and reduced post-harvest losses enhance its economic value by minimising food spoilage and reducing risks associated with market fluctuations (52).

The nutritional and health-promoting properties of finger millet have increased interest in expanding its cultivation and integrating its products into mainstream food systems, particularly in countries such as Uganda and Kenya (81). The growing demand for value-added finger millet products presents opportunities for commercialisation and market expansion. Increased commercial production could generate substantial socio-economic benefits in producing regions through employment creation across the value chain, particularly in labour-intensive activities such as harvesting, threshing, and dehulling. Furthermore, improved market linkages and value addition could increase the availability and accessibility of nutrient-rich finger millet products, thereby contributing to food and nutrition security amongst both rural and urban populations.

**Figure 4:**
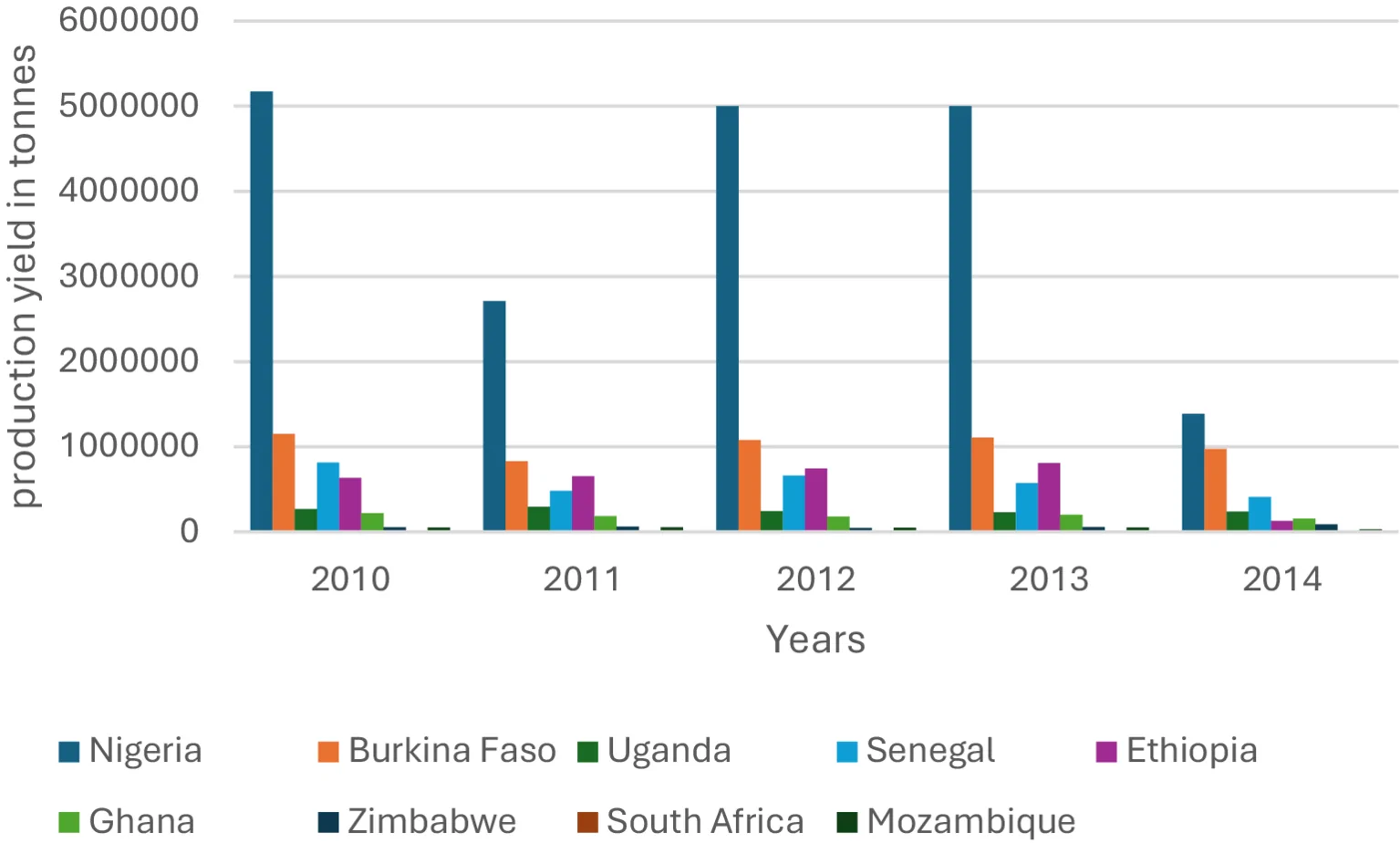
Millet production trends in selected sub-Saharan African countries over a four-year period (adapted from (81)). NB: Although the production statistics are more than a decade old, they are included to illustrate historical production patterns, particularly because recent country-level data on millet production trends in Africa remain limited.

The lack of recent country-level production data for individual millet species, in contrast to well-documented staple crops such as maize and rice, represents a significant research and knowledge gap. For instance, Figure 4 indicates that Nigeria has the highest overall millet production amongst the selected countries; however, these aggregated statistics obscure species-specific production trends (82). This is particularly evident for finger millet, where Ethiopia is the leading producer in Africa. The absence of updated detailed reporting on individual millet species reflects their marginalisation within formal agricultural monitoring systems and highlights the need for improved data collection, classification, and reporting frameworks. Consequently, this limitation affects accurate evaluation of production trends, economic contributions, and the development of targeted policies to support these underutilised crops, despite their importance in food security and climate resilience.

Globally, finger millet production remains difficult to quantify because FAO statistics aggregate all millet species into a single category, limiting the availability of species-specific production estimates. However, available country-level data indicate that India is the leading producer of finger millet, with an annual production of approximately 1.8 million tonnes, followed by Ethiopia (1.2 million tonnes), Nepal (0.31 million tonnes), Uganda (0.20 million tonnes), and Tanzania (0.10 million tonnes) (155). These disparities in reporting further emphasise the need for improved documentation of individual millet species to better understand their contribution to global food systems.

## 8 Nutritional significance and phytochemical profile

Finger millet possesses a rich mineral and micronutrient profile, highlighting its potential contribution to addressing food and nutrition insecurity in Africa compared with many conventional cereal crops (153). It is particularly recognised for its exceptionally high calcium content, ranging from 240 to 344 mg/100 g (Table 1) (83). This high calcium concentration is important for bone development and may help reduce the risk of osteoporosis (149), particularly in resource-limited communities where micronutrient deficiencies are prevalent (84). In addition, finger millet contains substantial amounts of magnesium (130–188.83 mg/100 g) and potassium (350–500 mg/100 g), minerals that contribute to metabolic regulation and cardiovascular health (84).

**Figure 5:**
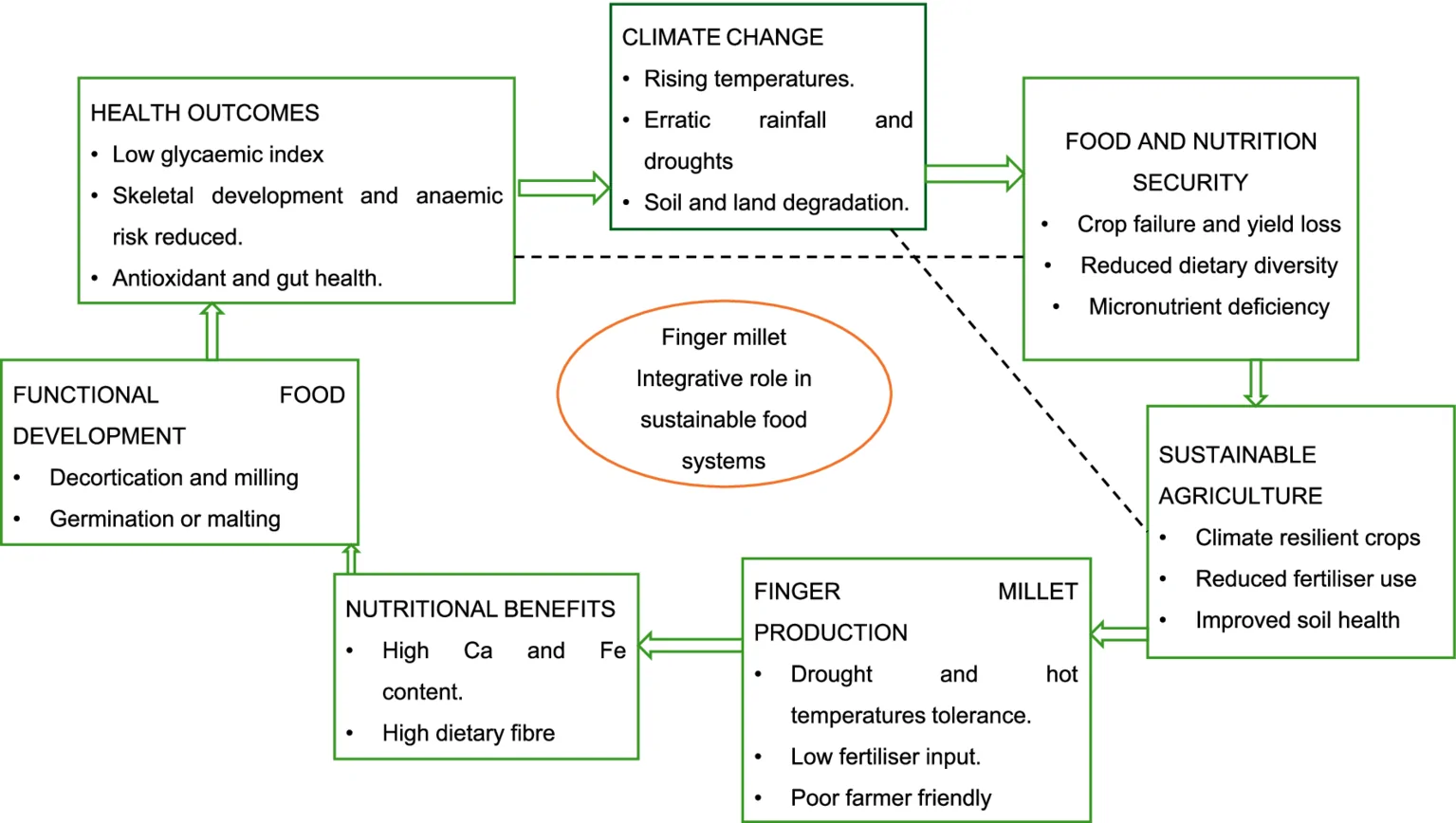
Conceptual framework that illustrates the integrative role of finger millet in sustainable food systems.

Millets are rich in complex carbohydrates that provide sustained energy through gradual digestion and absorption (18). Compared with commonly consumed cereals such as rice and maize, finger millet generally contains higher concentrations of several micronutrients, making it a valuable source of dietary energy and essential nutrients. Although its protein content is lower than that of some other millets, including foxtail and pearl millet (Table 2), its nutritional quality can be improved through traditional processing methods such as fermentation, malting, and sprouting, which enhance nutrient availability and digestibility (85). Furthermore, its high dietary fibre content (3.6–11.5%) supports digestive health, improves bowel function, and may reduce the risk of metabolic disorders. These nutritional benefits highlight the importance of promoting finger millet consumption through the development of diverse, acceptable, and value-added food products (Figure 5).

Beyond its nutritional attributes, finger millet’s ability to tolerate harsh environmental conditions, including drought and poor soils, whilst producing stable yields with minimal external inputs, enhances its suitability for smallholder farmers in marginal environments. Combined with its nutritional value and economic potential, these characteristics position finger millet as a promising crop for current and future food and nutrition security interventions (Table 5).

**Table 5:** Some finger millet phenolic compound compositions and their associated bioactivity.

Phenolic compound classes	Major compounds	Key structural types	Bioactivity	References
Phenolic acids	Protocatechuic acid, Protocatechuic aldehyde	Hydroxybenzoic acids	Antioxidant and anti-microbial	(88, 92)
Flavan-3-ols	Catechin, Epicatechin, Catechin derivatives	Monomers and oligomers	Anti-cancer	(92, 130)
Procyanidins	Procyanidin dimers	Condensed tannins	Antioxidant and antimicrobial	(92)
Flavonols	Quercetin, Quercetin glycosides (rutinoside, dihexoside)	Glycosylated flavonols	Anti-cancer	(92, 130)
Flavones	Apigenin derivatives (C- and O-glycosides)	C-glycosyl flavones	Anti-cancer, anti-ageing and cardioprotective	(92, 130)
Mixed flavonoid glycosides	Apigenin–pentosyl–hexoside derivatives	Complex glycosides	Antioxidant and metabolic effects	(92)

In addition to its role as a human food source, finger millet has several household applications that contribute to its multifunctionality. The grain is used as livestock feed, whilst milling produces bran that can be incorporated into diets for poultry and pigs (86). After grain harvesting, dried leaves and straw are utilised as feed resources for livestock, including cattle, sheep, and goats (86). Furthermore, finger millet is traditionally processed into fermented alcoholic and non-alcoholic beverages in various African communities, contributing to cultural practises and local food systems (81).

### 8.1 Bioactive compound profile and biological activities

Finger millet’s nutritional and health-promoting potential is largely attributed to its dense and diverse phytochemical profile, which is more complex than that of many major cereal crops. Phytochemicals are biologically active compounds that contribute to physiological functions and provide various health benefits (87). Metabolomic profiling of finger millet cultivated in Jammu and Kashmir identified 53 phenolic compounds, with coumarin and eugenol being amongst the major constituents, and demonstrated that phenolic compounds contribute significantly to the crop’s antioxidant capacity (88). Processing techniques such as germination and fermentation further enhance antioxidant activity by increasing phenolic content, although the extent of bioactivity is influenced by processing methods (89). Germination activates antioxidant defence mechanisms, resulting in increased production of phenolic compounds, flavonoids, tannins, and vitamins C and E, whilst promoting the synthesis of bioactive peptides with free radical scavenging and lipid oxidation-inhibiting properties (90).

Polyphenolic compounds are predominantly concentrated in the outer layers of finger millet grains, particularly the seed coat. Extraction and characterisation of seed coat polyphenols using acidified methanol revealed the presence of benzoic acid derivatives, cinnamic acid derivatives, and the flavonoid quercetin (91). A study of four finger millet varieties from northern Malawi reported free phenolic contents ranging from 114.43 to 179.19 mg ferulic acid equivalents (FAE)/100 g and bound phenolic contents ranging from 58.27 to 123.23 mg FAE/100 g. Twenty phenolic compounds were identified in the free fraction and seventeen in the bound fraction, with ferulic acid being the predominant bound phenolic compound (81). Phenolic concentration also varies amongst finger millet varieties, with coloured varieties generally exhibiting higher levels than non-coloured varieties (92). This association between seed pigmentation and polyphenolic abundance highlights the potential value of darker-seeded landraces in breeding programmes targeting enhanced phenolic content and health-promoting properties.

Flavonoids represent an important class of phenolic compounds in finger millet, with LC–MS-based profiling identifying them as major contributors to the seed’s phenolic composition (88). Catechin and epicatechin are amongst the predominant extractable flavonoids, whereas ferulic acid remains the major bound phenolic compound. Beyond genetic variation, flavonoid accumulation can be influenced by agronomic and biotechnological approaches. For example, methyl jasmonate elicitation in finger millet sprouts reduced sprout elongation whilst significantly enhancing flavonoid and phenolic biosynthesis. This response was associated with increased oxidative signalling, including 1.84-fold and 1.70-fold increases in hydrogen peroxide and superoxide anion concentrations, respectively, alongside enhanced catalase and peroxidase activity compared with untreated controls (93). These findings demonstrate that the polyphenolic profile of finger millet is dynamic and can potentially be manipulated through targeted cultivation and processing strategies to develop flavonoid-enriched functional food products.

## 9 Anti-nutritional factors, processing, and bioavailability

### 9.1 Identity and distribution of finger millet’s anti-nutritional compounds

Despite its favourable mineral composition, finger millet contains several anti-nutritional factors (ANFs) that may limit the nutritional benefits of its mineral content. The major anti-nutrients reported in millets include phytates, tannins, oxalic acid, enzyme inhibitors, and saponins, which can bind essential minerals such as iron, calcium, zinc, and magnesium, thereby reducing their bioavailability (94). Consequently, although finger millet is a rich source of minerals, the presence of these compounds can interfere with mineral absorption and utilisation by the body (95). In addition to mineral chelation, ANFs can negatively influence nutrient digestion and physiological functions. Phytic acid reduces mineral availability, enzyme inhibitors interfere with protein and carbohydrate digestion, and certain phenolic compounds may reduce protein digestibility. Excessive intake of these compounds has also been associated with gastrointestinal discomfort, impaired enzyme activity, and disruptions in thyroid hormone function (94).

Anti-nutritional factors and nutrients are not evenly distributed throughout the grain but are concentrated in specific anatomical regions. In millets, iron is predominantly located in the aleurone layer, which is often removed during milling (96). Similarly, phenolic compounds are mainly concentrated in the pericarp, hull, aleurone layer, and endosperm (94). This co-localisation of minerals and anti-nutrients within peripheral grain tissues explains why processing methods that remove the seed coat may simultaneously reduce both anti-nutritional compounds and valuable nutrients.

#### 9.1.1 Mechanisms of action: how anti-nutrients impair mineral absorption

##### 9.1.1.1 Phytic acid (phytate)

Phytic acid is a phosphorus storage compound that readily chelates essential mineral cations, forming complexes known as phytates or phytin, which are primarily responsible for its anti-nutritional effects (150). A study involving women with iron deficiency reported that iron bioavailability from ragi (finger millet) was only 4.6%, compared with 8.3% from white rice and 11.2% from whole wheat atta flour. This reduced bioavailability was attributed to the relatively higher phytic acid and tannin content of finger millet (Reddy et al., 2022). These findings highlight that, despite its high iron content, the nutritional benefit of finger millet may be limited by poor mineral bioavailability, challenging the assumption that its mineral abundance directly translates into superior iron nutrition compared with other cereals.

##### 9.1.1.2 Tannins

Tannins exert anti-nutritional effects through mechanisms that differ from those of phytates, primarily by reducing protein digestibility and limiting iron bioavailability. The low iron bioavailability observed in brown finger millet varieties has been associated with their higher tannin content (97). In addition, tannins contribute to a bitter taste and reduce the apparent digestibility of proteins, potentially affecting consumer acceptability and nutritional value (97). Although grain colour may serve as a useful preliminary indicator of tannin levels, particularly for low-cost screening in breeding programmes, analytical confirmation remains necessary because colour alone cannot reliably quantify tannin content.

##### 9.1.1.3 Polyphenols and oxalates

Polyphenols can reduce micronutrient absorption and bioavailability through their metal-chelating properties and may also influence grain storage stability (98). Oxalate, reported at approximately 0.27% in finger millet, can interfere with nutrient utilisation by forming complexes with proteins and reducing calcium and magnesium absorption. It is also associated with kidney stone formation; however, consumption of oxalate-containing foods is generally considered safe for healthy individuals, whilst those with impaired gastrointestinal or renal function may experience greater sensitivity (99).

#### 9.1.2 Anti-nutritional reducing techniques

The most recent verifiable data from Bhuvaneshwari et al. (100) report tannin reduction following a combined soaking–germination treatment; however, the specific contribution of soaking alone, independent of the enzymatic effects associated with germination, remains unclear. Across the major processing methods used for finger millet preparation, including soaking and germination, decortication, fermentation, and cooking, significant knowledge gaps remain regarding their comparative effectiveness in reducing anti-nutritional compounds. Furthermore, variations in reporting units, cultivars, and baseline polyphenol or tannin concentrations limit direct comparisons of processing efficiency across studies.

## 10 Health-promoting properties of finger millet

Vegetables, fruits, nuts, and whole grains are recognised as important sources of phytochemicals with significant health benefits (87). Finger millet, although considered a minor millet, contains high concentrations of bioactive compounds, particularly polyphenols and dietary fibre (101). These compounds contribute to the potential role of finger millet in mitigating non-communicable diseases (NCDs), including obesity and diabetes (Table 6). Deep brown finger millet varieties contain polyphenol levels that exceed those of several conventional cereal crops, highlighting their potential functional food value (102). NCDs, also referred to as chronic diseases, account for more than 70% of global deaths and are strongly associated with lifestyle factors, including poor dietary patterns (103, 104).

**Table 6:** Finger millet functional foods and their health benefits.

Functional food category	Functional components	Health benefits	Processing methods		References
Whole grain foods	Dietary fibre, polyphenols, calcium, iron	Improved digestion, antioxidant activity, glycaemic control, and bone development	Milling, roasting, cooking	Whole finger millet flour, porridge	(137)
Fermented foods	Probiotics, bioactive peptides, enhanced phenolics	Improved gut health, enhanced mineral bioavailability, reduced antinutrients	Fermentation, malting	Fermented porridge, traditional beverages	(26, 123)
Milling and malting process	Bioavailable minerals, amino acids, enzymes	Enhanced digestibility and nutrient absorption	Germination, drying, milling	Malted finger millet flour	(26, 138)
Snack foods	Polyphenols, dietary fibre	Healthier snack alternative with antioxidant properties	Extrusion, roasting, puffing	Extruded snacks and noodles	(139)
Weaning/infant foods	Calcium, iron, amino acids	Supports growth and bone development in infants	Malting, fermentation, cooking	Finger millet infant porridge	(140, 141)
Functional beverages	Phenolic compounds, probiotics	Gut health promotion and antioxidant activity	Fermentation	Finger millet probiotic drinks	(142)
Baked products	Fibre content and minerals content. Rich in calcium content.	Gluten free products	Milling and malting process	Bread, nankhatai, biscuits and muffins	(123)

### 10.1 Diabetes/glycaemic control

Finger millet bioactive compounds, particularly seed coat phenolics, have demonstrated potential anti-diabetic properties through inhibition of key carbohydrate-digesting enzymes, including intestinal pancreatic *α*-amylase and α-glucosidase, which regulate glucose release into the bloodstream (105). However, much of the mechanistic evidence is derived from *in vitro* enzyme inhibition studies rather than whole-food interventions. Finger millet seed coat phenolics strongly inhibited α-glucosidase and pancreatic α-amylase, with IC50 values of 16.9 and 23.5 μg phenolics, respectively, whilst kinetic analysis indicated a non-competitive mode of inhibition (106). Similarly, finger millet extracts demonstrated lower IC50 values for both enzymes compared with the pharmaceutical drug acarbose (107). Although these findings provide strong biochemical evidence, they represent isolated compound activity under controlled laboratory conditions and do not necessarily translate directly to glucose regulation in humans.

Animal studies provide additional evidence supporting these mechanisms. Finger millet seed coat material containing 11.2% polyphenols reduced fasting blood glucose, serum urea, serum creatinine, serum cholesterol, and HbA1c levels in diabetic rats (106). Human evidence remains limited but emerging. A single-blind randomised controlled crossover trial reported that finger millet muffins reduced peak glycaemic response and insulin response area under the curve in prediabetic participants, although the overall reduction in glycaemic response was not statistically significant (108). Furthermore, a systematic review of six randomised controlled trials found that multigrain formulations containing finger millet, together with other grains such as oats and maize, reduced HbA1c and LDL-cholesterol levels in individuals with type 2 diabetes. However, the authors emphasised the need for better-controlled and multi-ethnic clinical trials (109). Therefore, current evidence supports the potential role of finger millet in glycaemic management, but insufficient evidence exists to conclusively claim that finger millet consumption prevents diabetes.

### 10.2 Wound healing

Individuals with diabetes often experience impaired wound healing due to compromised physiological processes, including reduced nerve growth factor activity and increased oxidative stress. Animal studies suggest that finger millet-derived compounds may improve wound healing by enhancing nerve growth factor production and antioxidant capacity (101). This is supported by observations of increased wound contraction rates comparable to non-diabetic controls in diabetic animal models, indicating potential benefits in tissue repair under metabolic stress (14). However, these findings require validation through clinical studies in diabetic patients.

### 10.3 Obesity/weight management

Finger millet has been proposed as a potential contributor to weight management due to its nutritional composition and bioactive compounds (148). Its tryptophan content has been suggested to influence appetite regulation pathways; however, direct human evidence supporting this effect in relation to finger millet consumption remains limited (110). Current evidence for tryptophan-mediated appetite regulation is derived mainly from animal studies, whilst most in vitro investigations have focused on carbohydrate-digesting enzyme inhibition rather than obesity-specific outcomes (111).

Animal studies provide preliminary evidence supporting the role for finger millet in weight regulation. A 12-week high-fat-diet mouse study demonstrated that finger millet bran supplementation reduced body weight gain, improved lipid profiles and inflammatory status, alleviated oxidative stress, and favourably altered gut microbiota composition (112). Similarly, finger millet flour supplementation reduced body weight gain and improved lipid parameters in rodents compared with a high-carbohydrate diet alone (113). Collectively, the nutritional composition and bioactive compounds of finger millet support its potential as a functional crop for improving dietary quality and contributing to food and nutrition security.

### 10.4 Anaemia

Beyond its role in metabolic health, finger millet’s mineral composition, particularly its iron content, suggests potential applications in addressing micronutrient deficiencies such as anaemia. Anaemia remains a significant public health challenge in Africa, particularly amongst women of reproductive age and children under 5 years (114). During pregnancy, anaemia increases the risk of adverse outcomes, including premature birth and low birth weight, whilst in children it can impair growth, development, and quality of life (115). Iron deficiency, often associated with inadequate dietary intake and broader socio-economic factors, remains a major contributor to anaemia (116). Therefore, incorporation of iron-rich foods such as finger millet into diets may contribute to strategies aimed at reducing iron deficiency; however, the impact is dependent on improving iron bioavailability through appropriate processing methods.

## 11 Traditional uses, functional foods, and value-added products

Finger millet can be used in traditional ways or in enhanced ways to add value for household consumption or to generate profit for households. Several traditional processing methods are available and important for proper utilisation of grains, for example, malting, soaking, cooking, fermentation, and popping (15). The different processing methods are essential for significantly reducing antinutrients and their effects in the grains, making them fit for human consumption (117). From the onset of its domestication, finger millet has been used for weaning food for babies from 6 months of age. It is fed to babies in the form of a porridge from sprouted flour (118). Finger millet is an ideal food for babies due to its high content of calcium and proteins (119).

### 11.1 Traditional fermented beverages

Finger millet is widely used in the production of traditional fermented beverages across the African continent where it serves as an important raw ingredient for both alcoholic and non-alcoholic beverages. These beverages are rooted in indigenous food systems and are consumed as staple a staple drink, refreshments, complementary foods and during cultural and ceremonial events (120). The fermentation process improves the sensory properties, digestibility and shelf life of finger millet products whilst enhancing mineral bioavailability through the degradation of antinutritional compounds. Additionally, fermentation promotes the production of beneficial metabolites and micro-organisms that contribute to the functional and health promoting properties of these beverages (121). Table 7 summarises the diverse range of indigenous finger millet based fermented alcoholic and non-alcoholic beverages that are produced across Africa, reflecting the crop’s broad geographical distribution and its continued importance in traditional food systems (120).

**Table 7:** Common indigenous finger millet-based fermented beverages.

Product	Country of production
*Busa (liquid drink)	Egypt
Doro/ chikokiyana/ utshwala (colloidal thick alcoholic drink); mahewu, maheu, mageu (fermented non-alcoholic beverage). A sour porridge is also produced.	Zimbabwe
Mahewu, mageu (fermented non alchololic beverage); umqomboti (alcoholic beverage); ting (fermented sour thick porridge/pap)	South Africa
*Kwanu-Zaki (liquid drink)	Nigeria
*Merissa (alcoholic drink)	Sudan
Busaa (fermented beverage with fermentation time determining alcohol percentage)	Kenya
Obushera (non-alcoholic fermented beverage)	Uganda
Tella (typically alcoholic beverage)	Ethiopia

*Adapted from Ramashia et al. (81).

### 11.2 Functional foods

Growing recognition of the nutritional value of finger millet has stimulated innovations in processing and product development aimed at enhancing consumer acceptability and integration into modern diets (122, 123). Processing approaches such as cooking, germination, fermentation, grinding, and malting (Table 8) improve digestibility, reduce anti-nutritional factors such as tannins, and enhance the bioavailability of essential minerals, including calcium and iron. These methods therefore support the development of diverse, nutritious, and consumer-oriented finger millet products.

**Table 8:** Finger millet processing techniques, their mechanism and effect on the antinutrients.

Processing method	Effect on tannins	Effect on polyphenols	Mechanism	References
Cooking/roasting/heating	Reduces phenolic/tannin bioactivity	Cooking significantly reduce total phenolic content and radical-scavenging activity, opposite to fermentation/acidification	Thermal degradation of phenolic compounds and denaturation of binding proteins	(143)
Fermentation	Tannin reduced from 1.64 to 0.72–0.80 mg/g over 24–36 h with *L. plantarum*; phytic acid reduced 6.30% in finger millet specifically	Strain and substrate dependent; comparable in magnitude to germination	Microbial/enzymatic breakdown by lactic acid bacteria	(144, 145)
Soaking	Highest tannin reduction amongst five millets tested (2.07 mg/g) under combined 24 h soaking + 24 h germination	Not separately isolated from germination effect in this study	Leaching of soluble tannins into soak water, supplemented by germination-associated enzymatic activity	(100)
Germination (malting)	Tannins reduced by 54%	Phytate reduced by 14%; protein increased from 7.52 to 8.64 g/100 g	Endogenous phytase and polyphenol oxidase activity during sprouting	(146)
Decortication/dehulling	Tannins decreased with increased polishing time, though loss intensified with more aggressive polishing	Phytates also decreased with polishing time and grain moisture content	Physical removal of the aleurone/seed-coat layer, where antinutrients are concentrated	(52)

## 12 Future research directions and commercial opportunities

### 12.1 Limitations of the current literature on finger millet

A major limitation contributing to the underutilisation of finger millet is the limited availability of robust human clinical evidence supporting its nutritional and health benefits. Existing human studies have largely been conducted in single locations and over short periods, making it difficult to determine whether observed effects remain consistent across Africa’s diverse dietary and agroecological conditions. Multi-location and multi-season trials are therefore needed to establish how genotype, environment, and processing practises influence nutritional outcomes.

Another challenge is the lack of species-specific production data for finger millet. Production statistics are often aggregated with other millet species or cereals, limiting accurate assessment of production trends, economic contributions, and commercial potential. Furthermore, value-chain research in Africa has largely focused on East African countries through a limited number of ICRISAT-led studies, leaving commercialisation potential in other regions poorly understood. Similarly, although bioactive compounds such as ferulic acid-rich arabinoxylans (feraxans), ferulic acid, caffeic acid, and quercetin have been identified in finger millet, the biochemical mechanisms underlying their antioxidant and anti-inflammatory effects remain insufficiently characterised (89). Thus, current knowledge remains largely descriptive, with gaps in production data, commercialisation research, and mechanistic understanding limiting the translation of finger millet’s nutritional and economic potential into practical outcomes.

### 12.2 Priority areas for future research

Future research should focus on integrating crop improvement, sustainable production, nutrition, and value-chain development. Genomics-assisted breeding should build on drought-tolerant accessions such as GBK042094 and GBK043137, which demonstrate improved physiological responses to water stress, including higher relative water content, chlorophyll retention, and proline accumulation (124). Marker-assisted selection and biofortification strategies should combine climate resilience with improved mineral profiles whilst accounting for genotype-related variation in calcium, iron, zinc, and phytate content (82, 125).

Climate-smart cultivation studies should evaluate improved genotypes under smallholder production conditions in regions such as Kenya, Tanzania, Uganda, and Ethiopia, where production is constrained by climate stress, pests, and competition from maize (60, 62, 126). These efforts should be supported by affordable mechanisation technologies to address labour-intensive processing activities such as threshing, dehulling, and milling, particularly amongst small and medium-scale processors constrained by limited capital and equipment availability (65). In addition, product development should expand beyond traditional flour and porridge to include innovative fermented, fortified, and functional food products.

Consumer acceptance studies are also required to understand factors limiting urban adoption of finger millet, as consumption remains largely rural despite increasing demand with income growth (64). Such studies should guide product development, pricing, and marketing strategies whilst maintaining traditional consumer preferences. Larger multi-country nutritional intervention trials are also needed to validate health benefits beyond small-scale studies, such as the three-month Kenyan trial amongst children with cerebral palsy (127).

Finally, strengthening finger millet commercialisation requires addressing weak farmer–processor linkages, dependence on traders, and limited collective marketing structures documented in East Africa (65, 128). Improved farmer aggregation, contract-based sourcing, extension support, and gender-responsive seed systems (66) are essential for ensuring that research advances translate into sustainable production, market integration, and improved food and nutrition security.

### 12.3 Conceptual framework

The diagram explains the relationship in which climate change through rising temperatures, erratic rainfall and soil degradation worsens food and nutrition security. This encourages the adoption of sustainable, climate smart agricultural practises. Moreover, finger millet is drought and heat resistant and requires low fertiliser inputs positioning it as one of the key climate smart crops. Due to these reasons, finger millet is both environmentally and economically friendly more particularly for poor farmers. Its high nutritional content of calcium, iron, polyphenols, dietary fibre and low glycaemic index provides the basis for functional food development through different processing techniques such as decortification, germination and fermentation which collectively reduces antinutritional properties whilst enhancing mineral accessibility and absorption. Produced nutritional foods from finger millet in turn contribute to improved health outcomes including glycaemic control, reduced anaemic risk, improved skeletal development, improved gut health, and antioxidant status. Lastly, improved health outcomes improve food and nutrition security (dashed black lines), whilst sustainable agricultural practises contribute to climate change mitigation and adaptation (dashed black lines), this affirms the bidirectional self-reinforcing nature of finger millet’s contribution to climate resilience, nutrition security and sustainable food systems.

### 12.4 Conclusion and prospects

Finger millet contains a high carbohydrate content, making it an important source of dietary energy. Although its protein content is lower than that of other millets, such as foxtail and pearl millet, traditional processing methods, including fermentation, malting, and sprouting, can enhance its nutritional quality. Its dietary fibre content further contributes to digestive health and may support improved metabolic function. Combined with its ability to withstand drought, poor soil, and low-input production conditions, finger millet is a valuable crop for smallholder farmers in marginal environments. These characteristics position finger millet as a promising component of food and nutrition interventions by linking climate resilience, sustainable production, dietary diversification, and functional food development, as summarised in the conceptual framework below.
